# Combined angiotensin-converting enzyme and aminopeptidase inhibition for treatment of experimental ventilator-induced lung injury in mice

**DOI:** 10.3389/fphys.2023.1109452

**Published:** 2023-03-30

**Authors:** Xinjun Mao, Verena Tretter, Yi Zhu, Felix Kraft, Benjamin Vigl, Marko Poglitsch, Roman Ullrich, Dietmar Abraham, Katharina Krenn

**Affiliations:** ^1^ Department of Anesthesia, General Intensive Care and Pain Medicine, Medical University of Vienna, Vienna, Austria; ^2^ Department of Anesthesiology, The Affiliated Hospital of Youjiang Medical University for Nationalities, Baise, China; ^3^ Department of Anesthesiology, The Central Hospital of Wuhan, Tongji Medical College, Huazhong University of Science and Technology, Wuhan, China; ^4^ Alterras Therapeutics GmbH, Vienna, Austria; ^5^ Attoquant Diagnostics GmbH, Vienna, Austria; ^6^ Department of Anesthesiology and Intensive Care Medicine, AUVA Trauma Center Vienna, Vienna, Austria; ^7^ Center for Anatomy and Cell Biology, Medical University of Vienna, Vienna, Austria

**Keywords:** ventilator-induced lung injury, renin-angiotensin system, aminopeptidase, angiotensin-converting enzyme inhibitor, hypotension

## Abstract

**Introduction:** Ventilator-induced lung injury (VILI) may aggravate critical illness. Although angiotensin-converting enzyme (ACE) inhibition has beneficial effects in ventilator-induced lung injury, its clinical application is impeded by concomitant hypotension. We hypothesized that the aminopeptidase inhibitor ALT-00 may oppose the hypotension induced by an angiotensin-converting enzyme inhibitor, and that this combination would activate the alternative renin-angiotensin system (RAS) axis to counteract ventilator-induced lung injury.

**Methods:** In separate experiments, C57BL/6 mice were mechanically ventilated with low (LVT, 6 mL/kg) and high tidal volumes (HVT, 30 mL/kg) for 4 h or remained unventilated (sham). High tidal volume-ventilated mice were treated with lisinopril (0.15 μg/kg/min) ± ALT-00 at 2.7, 10 or 100 μg/kg/min. Blood pressure was recorded at baseline and after 4 h. Lung histology was evaluated for ventilator-induced lung injury and the angiotensin (Ang) metabolite profile in plasma (equilibrium levels of Ang I, Ang II, Ang III, Ang IV, Ang 1-7, and Ang 1-5) was measured with liquid chromatography tandem mass spectrometry at the end of the experiment. Angiotensin concentration-based markers for renin, angiotensin-converting enzyme and alternative renin-angiotensin system activities were calculated.

**Results:** High tidal volume-ventilated mice treated with lisinopril showed a significant drop in the mean arterial pressure at 4 h compared to baseline, which was prevented by adding ALT-00 at 10 and 100 μg/kg/min. Ang I, Ang II and Ang 1-7 plasma equilibrium levels were elevated in the high tidal volumes group versus the sham group. Lisinopril reduced Ang II and slightly increased Ang I and Ang 1-7 levels versus the untreated high tidal volumes group. Adding ALT-00 at 10 and 100 μg/kg/min increased Ang I and Ang 1-7 levels versus the high tidal volume group, and partly prevented the downregulation of Ang II levels caused by lisinopril. The histological lung injury score was higher in the high tidal volume group versus the sham and low tidal volume groups, and was attenuated by lisinopril ± ALT-00 at all dose levels.

**Conclusion:** Combined angiotensin-converting enzyme plus aminopeptidase inhibition prevented systemic hypotension and maintained the protective effect of lisinopril. In this study, a combination of lisinopril and ALT-00 at 10 μg/kg/min appeared to be the optimal approach, which may represent a promising strategy to counteract ventilator-induced lung injury that merits further exploration.

## 1 Introduction

Mechanical ventilation is an essential means of life support for patients in acute respiratory failure, e.g., due to acute respiratory distress syndrome (ARDS) (Ashbaugh et al., 1967). ARDS continues to be associated with high overall mortality of 35%–45% (Bellani et al., 2016), and a critical factor contributing to this mortality is ventilator-induced lung injury (VILI) (Matthay et al., 2003; Lanspa et al., 2019). Broader knowledge about the mechanisms underlying VILI in severely ill patients, especially those with respiratory failure, is the base of preventive strategies, most prominently lung-protective ventilation (Acute Respiratory Distress Syndrome et al., 2000).

There is general consensus that the main mechanisms of VILI include volutrauma (tissue stress resulting from alveolar overdistension with increasing transpulmonary pressure), atelectrauma (high local shear forces caused by repeated alveolar recruitment and de-recruitment), and biotrauma [inflammation and activation of the renin-angiotensin system (RAS)] (Gattinoni et al., 2010; Beitler et al., 2016; Curley et al., 2016; Wang et al., 2019). The first two factors are mechanical in nature. They stimulate leukocyte recruitment and cause chemokine/cytokine release, which results in regional and systemic inflammation (Slutsky and Tremblay, 1998; Thompson et al., 2017). In contrast, biotrauma as the third factor is non-mechanical and includes propagation of inflammation and activation of the RAS (Jerng et al., 2007; Krenn et al., 2021; Mao et al., 2022).

Although angiotensin-converting enzyme (ACE) inhibition has been shown to offer protection against VILI (Jerng et al., 2007; Jiang et al., 2007), its hypotensive effects impede clinical application. The ACE inhibitor captopril counteracted VILI in experimental studies (El-Sayed et al., 2020; Mao et al., 2022), while lisinopril has not yet been investigated in this context.

Aminopeptidase inhibitors have been used in phase I and II clinical trials for cancer treatment (Dennis et al., 2021) and as an experimental treatment to attenuate the pressor response induced by aminopeptidase A in the brain (Boitard et al., 2019; Gupta et al., 2021). The new drug candidate ALT-00 prevents N-terminal proteolytic degradation of angiotensin metabolites, including Ang 1-7, thereby activating the alternative RAS pathway (Trubacova, et al. manuscript in preparation).

We hypothesized that ALT-00 may oppose the hypotensive effects of lisinopril, and that a combination of both drugs would still result in sufficient RAS modification to prevent VILI. In this study, we investigated the effect of lisinopril, alone and in combination with ALT-00 at three different dose levels, on the RAS in VILI and observed the change in mean arterial pressure (MAP) during the experiment. The RAS profile was evaluated by measuring the equilibrium plasma concentrations of Ang I, Ang II, Ang III, Ang IV, Ang 1-7, and Ang 1-5 and calculation of angiotensin metabolite concentration-based markers of renin activity, ACE activity, and alternative RAS activation.

## 2 Materials and methods

### 2.1 Animal preparation

All experimental protocols were reviewed and approved by the Committee for Animal Experimentation of the Medical University of Vienna and the Austrian Ministry of Education, Science and Research (“Role of the renin-angiotensin system in long-term ventilator-induced lung injury: An experimental study in mice,” permit number 2020-0.009.485 on 21 January 2020). All experiments were performed in the research laboratories at the Center for Biomedical Research of the Medical University of Vienna, Vienna, Austria, in compliance with National Institutes of Health guidelines.

Healthy C57BL/6 mice of both sexes, 8–12 weeks of age, with a body weight ranging from 20 to 27 g were randomly subdivided into 7 groups (*n* = 14), denoted as sham group (unventilated controls), low tidal volume (LVT, 6 mL/kg) and high tidal volume group (HVT, 30 mL/kg), as well as HVT + lisinopril groups ± ALT-00 at dose levels of 2.7, 10 or 100 μg/kg/min. The animals were housed at a room temperature of 22°C ± 2°C, an air humidity of 55% ± 10%, a 12:12 h dark:light regime in Makrolon^®^ cages on softwood litter. Animals were anesthetized with 3% isoflurane for about 2–3 min, injected intraperitoneally (i.p.) with ketamine (80 mg/kg) and xylazine (6 mg/kg), and placed on a heating pad. A tracheostomy was performed, and the mice were connected to a rodent ventilator (VentElite, Harvard Apparatus, Boston, MA) in volume control mode with the following initial settings: In the LVT group with a VT of 6 mL/kg, positive end-expiratory pressure (PEEP) of 1 cm H_2_O and respiratory rate of 135 breaths/min, and in the HVT group with a VT of 30 mL/kg, PEEP of 0 cm H_2_O and respiratory rate of 40 breaths/min; the fraction of inspired oxygen FiO_2_ was 0.5 and I:E = 1:2 in both groups. A bolus dose of rocuronium 1 mg/kg was given i.p., to prevent dyssynchrony with the ventilator.

A polyethylene catheter was inserted into the right carotid artery for continuous MAP monitoring as well as for blood gas analysis and blood sampling at the end of the experiment. Another polyethylene catheter was inserted into the right jugular vein for intravenous fluid and drug administration. Solution 1 (S1) for fluid therapy contained 80% Ringer’s lactate and 20% glucose 5%, solution 2 (S2) for maintenance of anesthesia contained 0.9% saline with ketamine 5 mg/mL, xylazine 0.3 mg/mL, and rocuronium 0.05 mg/mL, and solution 3 (S3) for experimental therapy contained lisinopril 0.15 μg/kg/min (a dose eliciting significant shifts in systemic RAS in pharmacodynamic pilot experiments) ± ALT-00 at one of three different dose levels (2.7, 10, and 100 μg/kg/min). All of these solutions were infused *via* a syringe pump (Kent Scientific, Torrington, CT). The S1 infusion rate was set at 30 mL/kg/h for 45 min to permit hemodynamic stabilization and was subsequently reduced to 15 mL/kg/h to maintain hemodynamic stability during ventilation. The S2 infusion rate was set at 10 mL/kg/h throughout the 4 h period in which the mice were exposed to ventilation. The S3 infusion rate was set for lisinopril at 0.15 μg/kg/min, and for ALT-00 at 2.7, 10 or 100 μg/kg/min, respectively.

The tidal volumes and duration of the experiment were chosen based on results from pilot experiments to induce VILI in wild type mice with significant alteration in lung mechanics. During HVT ventilation, with PEEP = 0 and peak inspiratory pressure = 30 cm H_2_O, carbon dioxide (CO_2_) was added to the inspiratory gas to avoid hypocapnia. The added CO_2_ concentration was regulated to achieve an end-tidal CO_2_ of 30–40 mmHg (4.0–5.3 kPa) that was monitored with a capnograph type 340 (Hugo-Sachs Elektronik - Harvard Apparatus, March, Germany). This helped to avoid respiratory acidosis or alkalosis, which may otherwise have influenced inflammatory mediators or could have contributed to the pathophysiology of acute lung injury (Laffey and Kavanagh, 2002; Takeshita et al., 2003; Doerr et al., 2005; Mao et al., 2022). During mechanical ventilation, each group was also subjected to additional recruitment maneuvers (35 cm H_2_O, 5 s) every 60 min throughout the experiment.

### 2.2 Physiological measurements

All mice were connected to a Powerlab System to monitor the electrocardiogram, heart rate (HR), MAP, and body temperature throughout the experiment that lasted 5 h in total: One hour for animal preparation (anesthesia, tracheostomy, and insertion of catheters) and 4 h of ventilation.

Baseline hemodynamic data was recorded in all ventilated groups after surgical preparation and stabilization. ALT-00 (synthesized at the Technical University Graz, Institute of Organic Chemistry) and lisinopril (Sigma-Aldrich, St. Louis, MO) were solved in saline. This was followed by administering a continuous intravenous infusion of lisinopril at 0.15 μg/kg/min, alone or combined with ALT-00 at 2.7, 10, or 100 μg/kg/min for 4 h in the respective treatment groups.

The hemodynamic parameters were recorded at 4 h.

### 2.3 Sampling subgroups

Each experimental group (*n* = 14) was divided into two alternating sampling subgroups of *n* = 7 mice. Gender of the mice was alternated within sampling subgroups to achieve an equal distribution of males and females within each experimental group and sampling subgroup. In one sampling subgroup, the right lung was removed and used to measure the wet-to-dry weight ratio, while bronchoalveolar lavage fluid (BALF) was obtained from the left lungs and analyzed by ELISA. In the other subgroup of mice the left lungs were instilled with 4% formalin at a hydrostatic pressure of 20 cmH_2_O for 15 min, then removed and stored in 4% formalin for lung histology. Paraffin-embedded lung tissue was sectioned at 3.5 µm and stained with hematoxylin-eosin. No animals or data points were excluded from analysis. All analyses of the samples were performed in an observer blinded manner.

### 2.4 Blood sample collection

At the end of the experiment, 70 μL of blood was collected from the carotid artery catheter for blood gas analysis, and heparin plasma from another 0.6 mL of blood was collected after centrifugation at 3,000 rpm (4°C) for 10 min and stored at −80°C for measuring the RAS parameters.

### 2.5 RAS equilibrium analysis

The equilibrium levels of angiotensin metabolites (Ang I, Ang II, Ang 1-7, Ang 1-5) in murine heparinized plasma samples were quantified with liquid chromatography-tandem mass spectrometry (LC-MS/MS) performed at Attoquant Diagnostics (Vienna, Austria) using previously validated and described methods (Kovarik et al., 2015; Basu and Yogasundaram, 2017). The lower limits of quantification (LLOQ) for Ang I, Ang II, Ang III, Ang IV, Ang 1-7 and Ang 1-5 were 4, 2, 3, 2, 3 and 4 pmol/L, respectively. The plasma was analyzed following equilibration at 37°C for 30 min. Samples where stabilized by chaotropic inactivation and spiked with stable isotope-labeled internal standards for individual angiotensin metabolites. Subsequently these samples underwent C-18-based solid-phase-extraction and were analyzed with LC-MS/MS, using a reversed-phase analytical column compatible with a Xevo TQ-S triple quadruple mass spectrometer (Waters, Milford, MA). Internal standards were used to correct peptide concentrations for recovery efficiency of the sample preparation for each analyte in every sample. Analyte concentrations were calculated from in-run calibration curves and reported in pmol/L.

We calculated the Ang concentration-based markers PRA-S (Ang I + Ang II concentrations) (Burrello et al., 2020; Guo et al., 2020), ACE-S (ratio of Ang II/Ang I concentration) (Burrello et al., 2020) and ALT-S [(Ang 1-7+Ang1-5 concentrations)/(Ang I+Ang II+Ang 1-7+Ang 1-5 concentrations)] to evaluate renin activity, ACE activity and activation of the alternative RAS axis, respectively (Mao et al., 2022).

### 2.6 Plasma renin activity

Plasma renin activity (PRA) was measured with an LC-MS/MS-based Ang I generation assay (Burrello et al., 2020). Heparin plasma was supplemented with an Ang I-stabilizing inhibitor mix (Attoquant Diagnostics) in phosphate-buffered saline (Dulbecco’s PBS, pH 7.4, Sigma-Aldrich). Of these mixtures, one aliquot was put on ice, while the other was incubated at 37°C for 60 min. Next, samples were stabilized and LC-MS/MS-based quantification of Ang I was performed as described above. The PRA was calculated by subtracting Ang I levels measured in samples on ice from Ang I levels determined in incubated samples as an intrinsic Ang I formation rate given in (ng Ang I/mL/h). The LLOQ of this assay was 0.02 ng Ang I/mL/h.

### 2.7 Bronchoalveolar lavage fluid sampling

In one sampling group of animals (*n* = 7), after 4 h of ventilation, 3 aliquots of phosphate buffered saline, 30 mL/kg each, were instilled into the left lungs and withdrawn slowly 3 times with a BALF recovery of > 90%. The recovered fluid was centrifuged at 3,000 rpm (4°C) for 5 min. The supernatant from the first sample was stored at −80°C for the determination of inflammatory cytokines, including interleukin (IL)-1β, IL-6, keratinocyte-derived cytokine (KC/CXCL1), and macrophage inflammatory protein-2 (MIP2/CXCL2) using Duoset ELISA kits (R&D Systems; Biotechne, MN) according to the manufacturer’s instructions.

### 2.8 Histological analysis

Hematoxylin/eosin-stained lung tissue sections were graded in an observer-blinded fashion with the experimental acute lung injury score (Matute-Bello et al., 2011; Kulkarni et al., 2022). The score involved numbers of neutrophils, presence of hyaline membranes and proteinaceous debris as well as alveolar wall thickening, and was calculated as described in the workshop report of the American Thoracic Society published by Matute-Bello et al., in 2011.

### 2.9 Data analysis

The sample size was based on our previous experience with this design (Mao et al., 2022) and on results of pilot studies. Data were analyzed by one-way analysis of variance with Tukey posttest, using GraphPad Prism 8.0 (GraphPad software, San Diego, CA). A *p*-value < 0.05 was considered significant. For angiotensin peptide levels below the LLOQ, the respective LLOQ divided by the square root of two was used for calculations (Croghan and Egeghy, 2003; Antlanger et al., 2017).

## 3 Results

### 3.1 Cardiorespiratory variables

**FIGURE 1:**
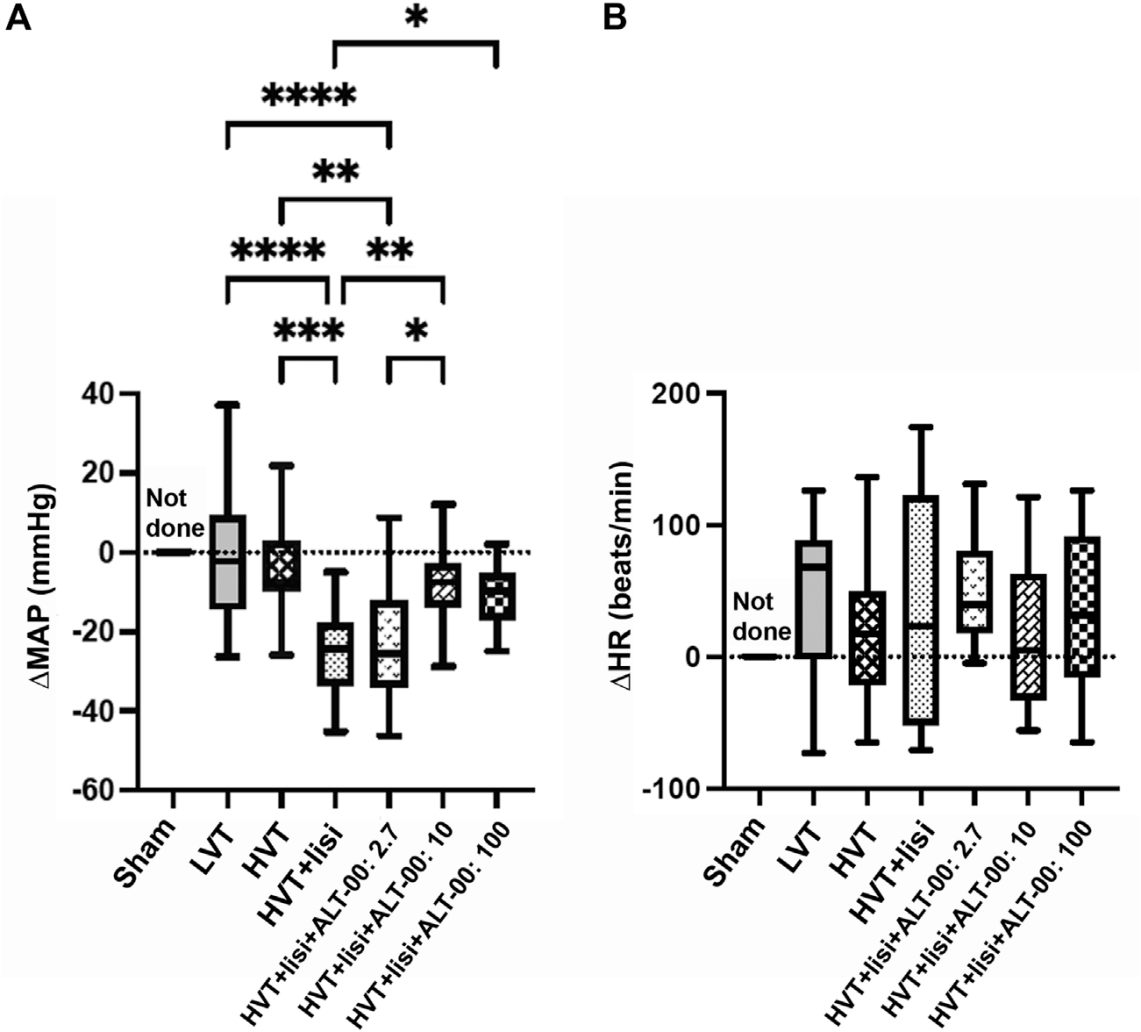
Hemodynamic parameters after 4 h of ventilation. **(A)** Mean arterial pressure (MAP): High tidal volume (HVT) ventilation + lisinopril (lisi) infusion substantially decreased the MAP shown here as a negative ΔMAP (=MAP at 4 h – MAP at baseline), which was prevented by additional treatment with ALT-00 at 10 and 100 μg/kg/min. With an ALT-00 dose of 2.7 μg/kg/min, the MAP did not change versus HVT + lisi and remained decreased versus the untreated HVT and LVT groups and the HVT group + lisi + ALT-00 at 10 μg/kg/min. **(B)** Heart rate (HR): ΔHR (= HR at 4 h – HR at baseline) showed no statistical differences between the groups, *n* = 14/group, hemodynamic measurements after 4 h not done in the sham group, ****p < 0.0001, ***p < 0.001, **p < 0.01, *p < 0.05, compared by one-way analysis of variance with Tukey posttest.

Hemodynamic parameters (MAP and HR) were monitored in all HVT groups for up to 4 h of ventilation. We calculated the ΔMAP = (MAP at 4 h)—(MAP at baseline) shown in Figure 1A, and the ΔHR = (HR at 4 h)—(HR at baseline) shown in Figure 1B. The ΔMAP was greatly decreased by treatment with lisinopril versus untreated LVT and HVT mice (*p* < 0.001), while additional treatment with ALT-00 at 10 and 100 μg/kg/min reduced the drop in MAP occurring in the group with HVT + lisinopril alone (*p* = 0.006 and *p* = 0.046, respectively). This protective effect of additional treatment with ALT-00 at 10 and 100 μg/kg/min on the drop in MAP induced by lisinopril was also observed in animals ventilated at LVT (Supplementary Figure S1). The ΔHR showed no differences between the groups.

**FIGURE 2:**
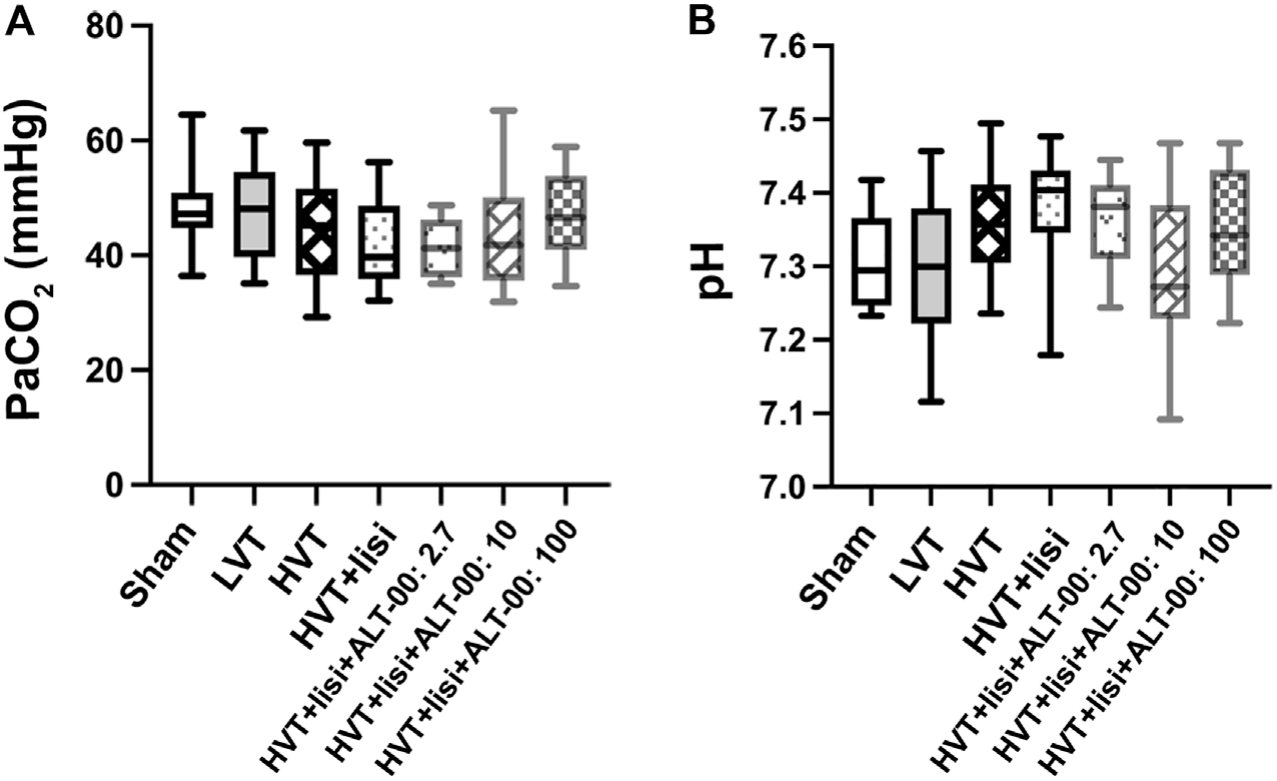
Acid base parameters. **(A)** The arterial partial pressure of carbon dioxide (PaCO_2_) and **(B)** the arterial pH were well maintained after 4 h without differences between the groups or the sham group at baseline, which indicated that tidal volume, respiratory rate and inspired CO_2_ were properly adjusted to maintain normocapnia; *n* = 14/group, baseline values are shown for the sham group, values after 4 h of ventilation for all other groups, tested by one-way analysis of variance with Tukey posttest.

The partial pressure of arterial carbon dioxide (PaCO_2_) and pH were well maintained after 4 h without significant differences between the groups (Figure 2).

### 3.2 Pulmonary edema and inflammation

**FIGURE 3:**
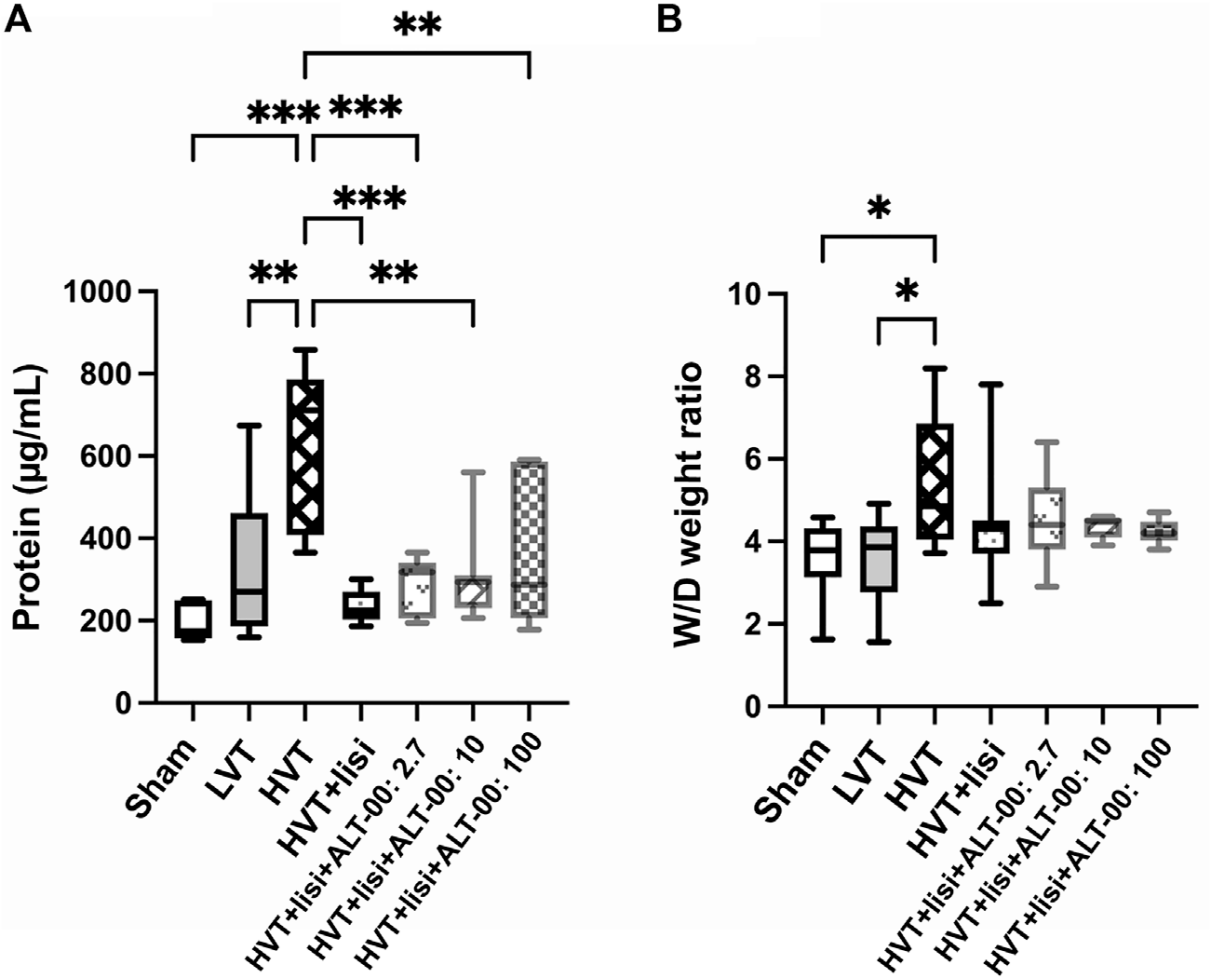
Assessment of pulmonary edema. **(A)** The protein content in the bronchoalveolar lavage fluid (BALF) was significantly elevated after high tidal volume (HVT) ventilation versus sham and low tidal volume (LVT) ventilation. Treatment with lisinopril (lisi) ± ALT-00 reduced the protein content versus the HVT group. **(B)** The lung wet-to-dry (W/D) weight ratio of the HVT group was increased versus the sham and LVT groups, while it was slightly reduced by lisi ± ALT-00 versus HVT controls. *n* = 7/group, ***p < 0.001, **p < 0.01, *p < 0.05, compared by one-way analysis of variance with Tukey posttest.

To assess the amount of pulmonary edema, the protein content in BALF and the lung wet-to-dry (W/D) weight ratios were measured. The BALF protein content (Figure 3A) in the HVT group was increased compared to the sham (*p* = 0.0006) and LVT (*p* = 0.002) groups. Treatment with lisinopril and ALT-00 decreased the BALF protein content compared to the untreated HVT group (all *p* < 0.01). The lung W/D weight ratio of the HVT group was increased versus the sham and LVT group (*p* = 0.044 and *p* = 0.045, respectively), and slightly reduced by treatment with lisinopril and ALT-00, as compared to HVT controls (Figure 3B).

**FIGURE 4:**
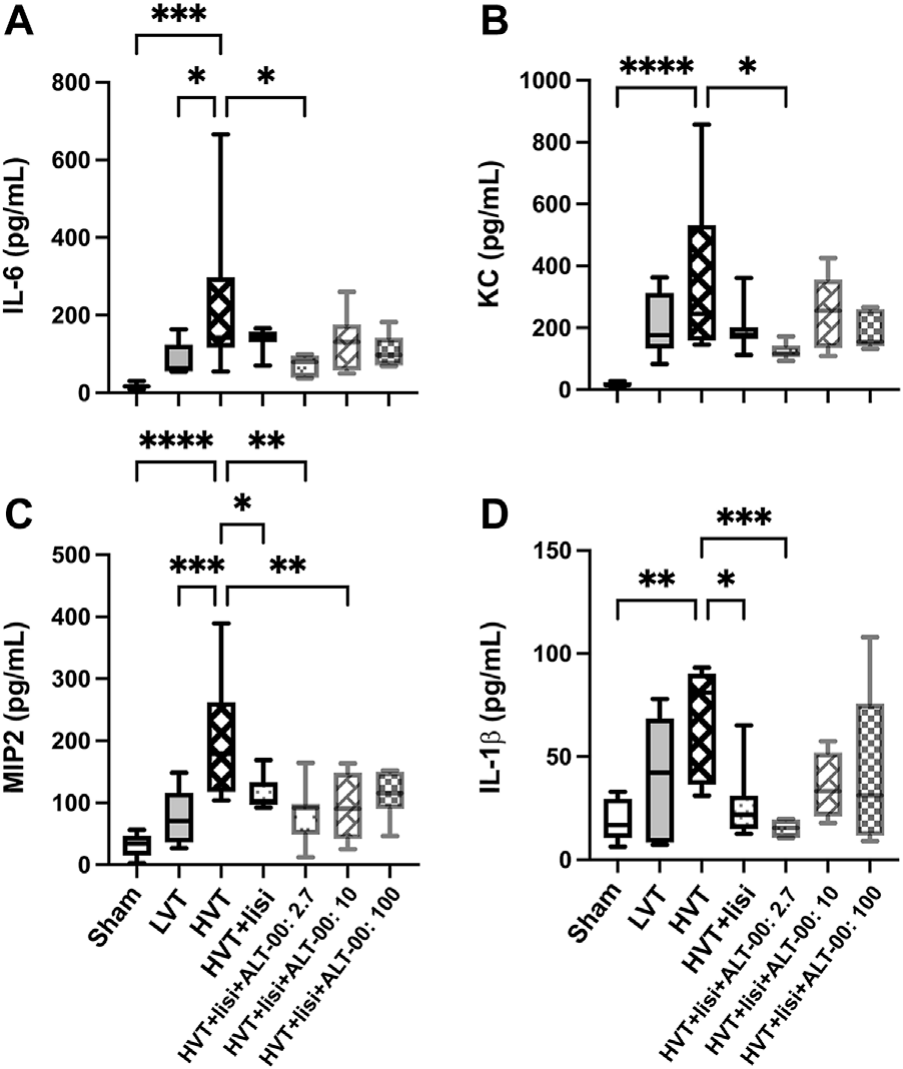
Assessment of inflammatory activation. **(A)** Interleukin (IL)-6 levels in bronchoalveolar lavage fluid (BALF) were significantly elevated in the high tidal volume (HVT) group versus the sham and the low tidal volume (LVT) group. Treatment with lisinopril (lisi) + ALT-00 (2.7 μg/kg/min) decreased IL-6 levels versus the HVT group. **(B)** The concentration of keratinocyte-derived cytokine (KC) in the HVT group was increased versus sham. Treatment with lisi +ALT-00 (2.7 μg/kg/min) decreased KC levels versus the HVT group. **(C)** The macrophage inflammatory protein-2 (MIP2) levels in the HVT group were highly increased versus the sham and the LVT group. Treatment with lisi ± ALT-00 (2.7 and 10 μg/kg/min) reduced MIP2 versus the HVT group. **(D)** The IL-1β levels in the HVT group were increased versus the sham group. Treatment with lisi ± ALT-00 (2.7 μg/kg/min) decreased IL-1β levels versus the untreated HVT group. *n* = 7/group, ****p < 0.0001, ***p < 0.001, **p < 0.01, *p < 0.05, compared by analysis of variance with Tukey posttest.

The evaluation of intra-alveolar inflammation with BALF concentrations of IL-6, KC, MIP2 and IL-1β showed similar patterns. Significant increases of all cytokines only occurred in the HVT group versus sham (*p* < 0.01), while treatment with lisinopril and ALT-00 at 2.7 and 10 μg/kg/min, but never at 100 μg/kg/min, reduced the BALF concentrations of individual inflammatory cytokines, as compared to the untreated HVT group (*p* < 0.05, Figures 4A–D). Specifically, lisinopril alone decreased MIP2 and IL-1β levels versus the untreated HVT group. With additional ALT-00 at 2.7 μg/kg/min IL-6, KC, MIP2 and IL-1β levels were decreased, and with ALT-00 at 10 μg/kg/min MIP2 levels were decreased, as compared to the untreated HVT group.

### 3.3 RAS profiles

**FIGURE 5:**
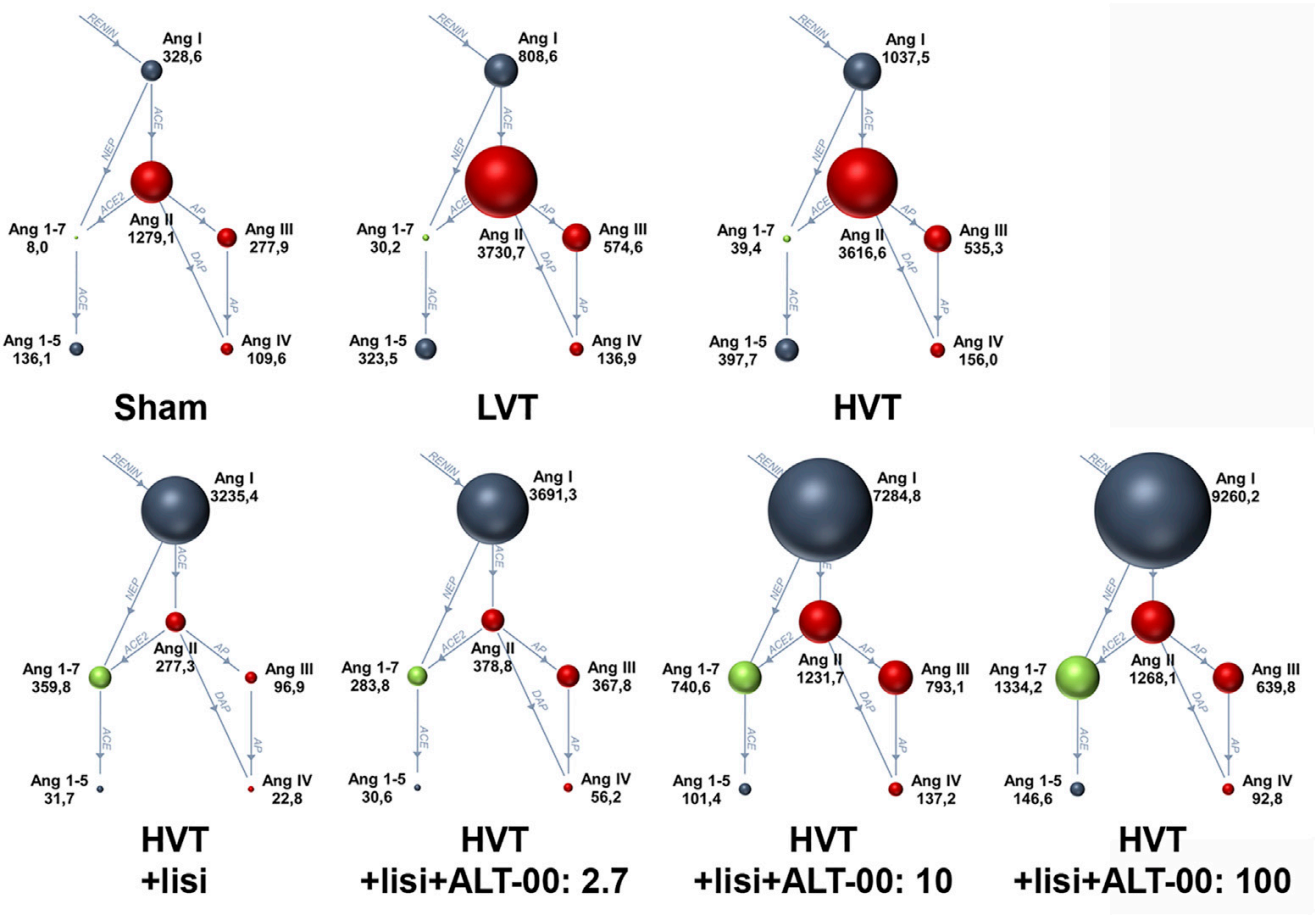
Renin-angiotensin system Fingerprints from equilibrium analysis of plasma samples of each experimental group. The median plasma concentrations of angiotensin metabolites in pmol/L in the RAS Fingerprints correspond to the sphere sizes, *n* = 7/group. Ang, Angiotensin; LVT, low tidal volume; HVT, high tidal volume; lisi, lisinopril, dose levels of ALT-00 in µg/kg/min.

**FIGURE 6:**
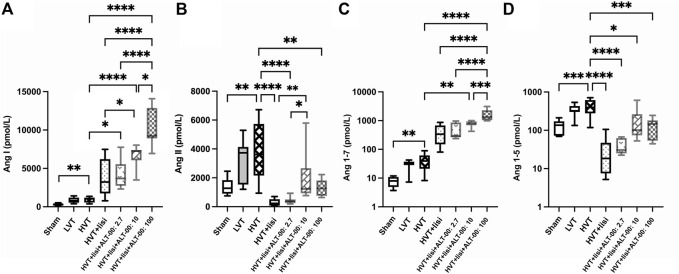
RAS equilibrium analysis. **(A)** Ang I concentrations were significantly elevated in the HVT group versus sham. Treatment with lisinopril (lisi) ± ALT-00 at all dose levels resulted in even higher levels of Ang I versus the HVT group. Moreover, lisi + ALT-00 at 100 μg/kg/min led to a greater increase of Ang I levels than dose levels of 2.7 or 10 μg/kg/min. **(B)** Ang II concentrations were significantly elevated in the HVT group versus sham. Treatment with lisi ± ALT-00 at all dose levels resulted in a drop of Ang II levels versus the HVT group. However, Ang II levels were higher in the HVT group treated with lisi + ALT-00 at 10 μg/kg/min than in the groups treated with lisi alone or in combination with ALT-00 at 2.7 μg/kg/min. **(C)** Ang 1-7 concentrations were elevated in the HVT group versus sham. Treatment with lisi + ALT-00 at dose levels of 10 and 100 μg/kg/min elevated Ang 1-7 levels even further versus the HVT group. Moreover, additional ALT-00 at 100 μg/kg/min increased Ang 1-7 levels more than 2.7 or 10 μg/kg/min, or lisi alone. **(D)** Ang 1-5 concentrations were increased in the HVT group versus sham. Treatment with lisi ± ALT-00 led to considerably lower Ang 1-5 levels versus the HVT group. ****p < 0.0001, ***p < 0.001, **p < 0.01, *p < 0.05, *n* = 7/group, compared by analysis of variance with Tukey posttest, logarithmic scale (log_10_) on the *y*-axis in **(C,D)**.

Figure 5 shows a summary of RAS Fingerprints from equilibrium analysis of plasma samples. These RAS Fingerprints include median equilibrium plasma levels of six angiotensin metabolites [Ang I, Ang II, Ang III (= Ang 2-8) and Ang IV (= Ang 3-8), as well as the alternative RAS metabolites Ang 1-7 and Ang 1-5]. The Ang I, Ang II, Ang 1-7 and Ang 1-5 plasma levels in the HVT group were increased compared to the sham group (*p* < 0.01, Figures 6A–D). Also LVT ventilation somewhat increased these angiotensin metabolite concentrations, but no significant differences compared to the sham group were found. Treatment with lisinopril ± ALT-00 decreased Ang II and Ang 1-5, and increased Ang I and Ang 1-7 concentrations in the respective HVT treatment groups versus the untreated HVT group (Figures 6A–D). The comparison of the HVT group receiving lisinopril with the groups receiving additional ALT-00 showed that Ang II concentrations were higher than in the group treated with lisinopril alone if ALT-00 was administered at 10 μg/kg/min (*p* = 0.008). Ang II concentrations in the group treated with lisinopril + ALT-00 at 10 μg/kg/min were also higher than in the group treated with lisinopril + ALT-00 at 2.7 μg/kg/min (*p* = 0.014). Ang II levels in the group treated with additional ALT-00 at 100 μg/kg/min were increased versus lisinopril alone, but without statistical significance. The angiotensin metabolite levels of mice ventilated at LVT under treatment with lisinopril and lisinopril + ALT-00 at 2.7 μg/kg/min are shown in Supplementary Figure S2.

**FIGURE 7:**
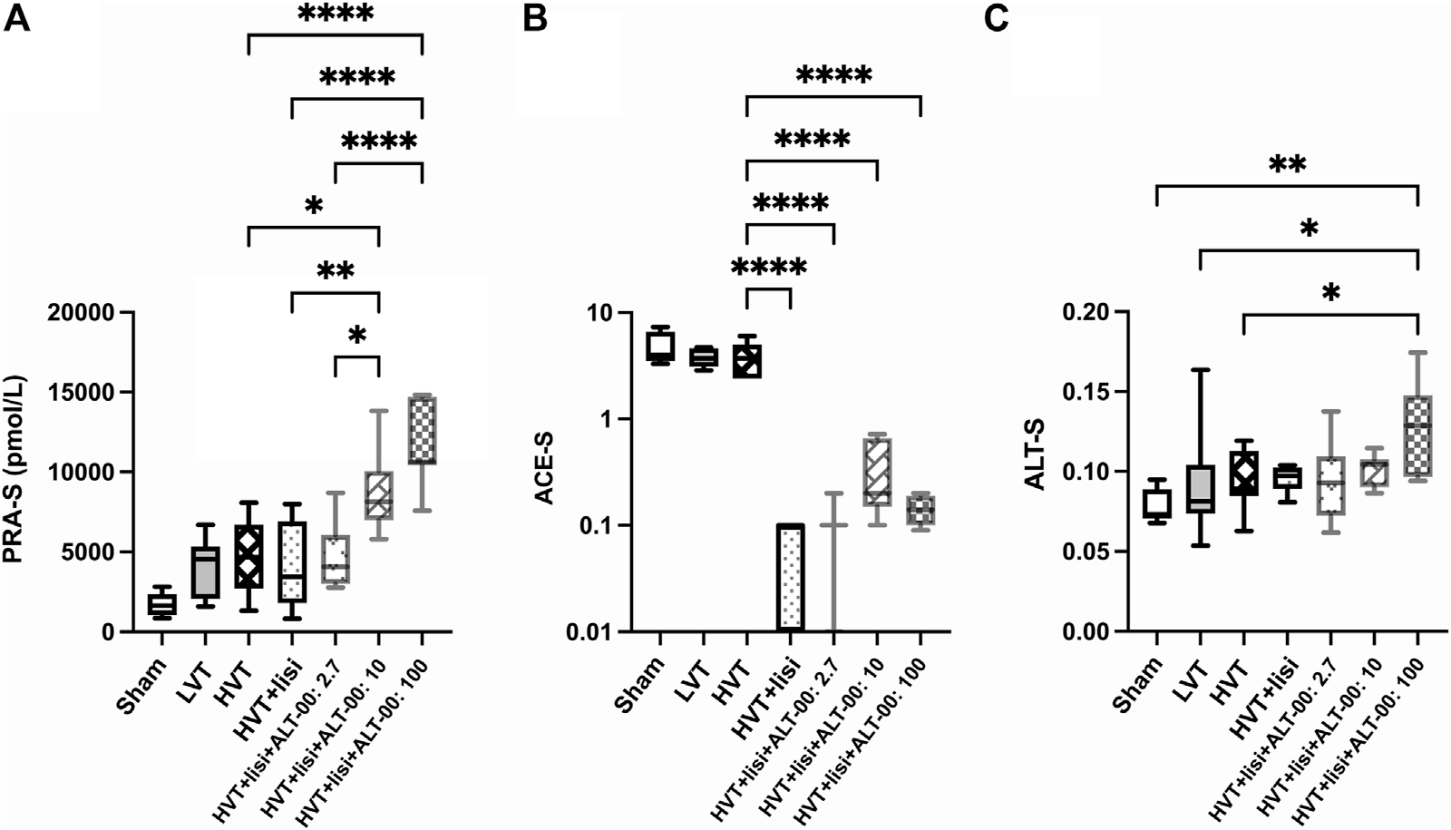
Angiotensin metabolite-based markers of angiotensin cleaving enzyme activities in plasma. **(A)** PRA-S (Ang I + Ang II) as a marker of plasma renin activity was increased following treatment with lisinopril (lisi) combined with ALT-00 at dose levels of 10 and 100 μg/kg/min versus the high tidal volume (HVT), HVT + lisi, and HVT + lisi + ALT-00 (2.7 μg/kg/min) groups. **(B)** ACE activity in plasma (Ang II/Ang I ratio, ACE-S) was downregulated in all groups treated with lisi ± ALT-00 versus the HVT group. **(C)** The alternative RAS activation marker ALT-S was increased by ALT-00 at a dose level of 100 μg/kg/min versus the sham, low tidal volume (LVT) and HVT groups. *n* = 7/group, ****p < 0.0001, **p < 0.01, *p < 0.05, compared by analysis of variance with Tukey posttest, logarithmic scale (log_10_) on the *y*-axis in **(B)**.

PRA-S as marker of plasma renin activity was increased following treatment with lisinopril + ALT-00 at 10 and 100 μg/kg/min versus the untreated HVT group, lisinopril treatment alone, and lisinopril + ALT-00 at the lowest dose level (*p* < 0.05 for ALT-00 at 10 μg/kg/min, *p* < 0.0001 for ALT-00 at 100 μg/kg/min; Figure 7A). A separate measurement of PRA in an Ang I generation assay showed that PRA was highest in the HVT group treated with lisinopril + ALT-0 at 100 μg/kg/min (*p* < 0.05 versus all other HVT groups), while there was no difference between the untreated HVT group and the other HVT groups receiving lisinopril and ALT-00 (data not shown). ACE activity in plasma (Ang II/Ang I ratio, ACE-S) was downregulated by lisinopril ± all dose levels of ALT-00 (Figure 7B), as compared to the untreated HVT group (all *p* < 0.0001). The alternative RAS activation marker ALT-S was increased by treatment with lisinopril combined with ALT-00 at 100 μg/kg/min (Figure 7C) versus the sham, LVT and HVT groups (*p* = 0.001, *p* = 0.030 and *p* = 0.042, respectively).

### 3.4 Lung histology

**FIGURE 8:**
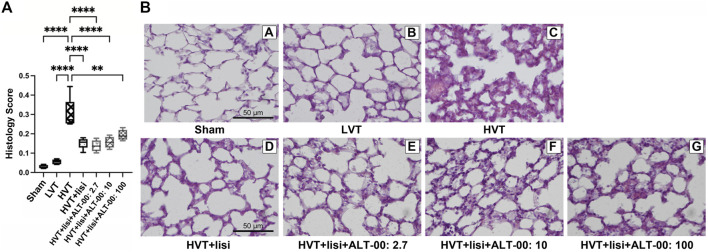
Histological lung injury score and representative histological images. **(A)** The lung histology scores for acute lung injury were upregulated after high tidal volume (HVT) ventilation. The histology score of the HVT group was increased versus the sham and low tidal volume (LVT) groups (****p < 0.0001), and was improved by lisinopril (lisi) ± ALT-00 (****p < 0.0001, **p = 0.002). *n* = 7/group, compared by analysis of variance with Tukey posttest. **(B)** Representative hematoxylin-eosin-stained histological images of each group, magnification ×40: The sham group (B/A) showed normal lung tissue structures and morphology. In the LVT group (B/B) the lung tissue sections showed a few hyaline membranes and neutrophils. The HVT group (B/C) showed neutrophil infiltration in the alveolar and interstitial space, extensive diffuse congestion with proteinaceous debris in the airspaces and alveolar septal thickening. In the group (B/D) with HVT ventilation treated with lisinopril (lisi) a few patches of alveolar septal thickening and proteinaceous debris in the airspaces were observed. The histological appearance in the groups treated with ALT-00 at (B/E) 2.7, (B/F) 10 and (B/G) 100 μg/kg/min was similar to the group treated with lisi alone.

The histological lung injury score was highest in the HVT group (*p* < 0.0001 versus sham and LVT group, Figure 8A), and was reduced by treatment with lisinopril ± ALT-00 (*p* < 0.01). Exemplary images of lung histology from each experimental group are shown in Figure 8B. The sham group showed normal lung tissue structures and morphology (Figure 8B/A). In the LVT group, the histological appearance resembled that of the sham group with a few hyaline membranes and neutrophils (Figure 8B/B). The HVT group showed neutrophil infiltration in the alveolar and interstitial space, alveolar septal thickening, and extensive diffuse congestion with proteinaceous debris in the airspaces (Figure 8B/C). In the HVT group receiving lisinopril a few patches of alveolar septal thickening and proteinaceous debris in the airspaces were observed (Figure 8B/D). Combined treatment with lisinopril and ALT-00 at all three dose levels led to a similar histological pattern as with lisinopril alone (Figure 8B/E–G).

## 4 Discussion

In this experimental study, we investigated the hemodynamic effects and the VILI prevention potential of a combined drug treatment using an ACE inhibitor (lisinopril) and three different dose levels of a new aminopeptidase inhibitor (ALT-00) in mice. Our main findings included the following: First, combined treatment with lisinopril and ALT-00 at dose levels of 10 and 100 μg/kg/min during HVT ventilation prevented the decrease in MAP after 4 h caused by lisinopril. Second, the combination of lisinopril with ALT-00 dose-dependently increased Ang I and Ang 1-7 plasma levels, and dose levels of 10 and 100 μg/kg/min partly stabilized Ang II levels that were suppressed by lisinopril alone or in combination with 2.7 μg/kg/min of ALT-00. This may have prevented hypotension after 4 h, while higher Ang 1-7 levels were evidence of anti-inflammatory actions mediated by lisinopril in combination with ALT-00. Third, ALT-00 at dose levels of 10 and 100 μg/kg/min increased PRA-S and at 100 μg/kg/min also PRA and ALT-S, which confirmed that the combination of lisinopril and ALT-00 activated the systemic classical and alternative RAS pathways in VILI. Fourth, the beneficial effects of lisinopril on the histologic lung injury score were maintained in the groups receiving additional ALT-00, although there was no significant reduction of any of the inflammation markers at the ALT-00 dose of 100 μg/kg/min.

Ang II is a key mediator for the inflammatory cascade that induces dose-dependent apoptosis of alveolar epithelial cells interacting with Angiotensin II type 1 receptor (AT1R) (Wang et al., 2019). Ang II levels are also increased in models of VILI, and inhibitors of ACE and AT1R that reduce Ang II levels or prevent activation of AT1R have been applied to counteract VILI in multiple experimental settings (Jerng et al., 2007; Jiang et al., 2007; Wösten-van Asperen et al., 2008; Mao et al., 2022). The desired effects are a decrease in Ang II-mediated signaling and an activation of the alternative RAS axis, both conveying anti-inflammatory effects (Santos et al., 2018). To date, the ACE inhibitor captopril has been most frequently studied in VILI (Jerng et al., 2007; Jiang et al., 2007; Wösten-van Asperen et al., 2008; Mao et al., 2022) and in other settings of experimental lung injury. It reduced airway hyperreactivity induced by hepatic ischemia-reperfusion injury by decreasing TNF-α levels in tracheal tissue (El-Sayed et al., 2020), and protected against acute pancreatitis-associated lung injury by inhibiting Ang II (Yu et al., 2016). Our study, to our knowledge, was the first to study lisinopril in VILI specifically. Only one study focusing on the local pulmonary RAS and lung injury investigated lisinopril treatment in neonatal murine lungs injured with bleomycin, where it decreased expression of angiotensinogen (Abdelwahab et al., 2016). In other experimental settings, lisinopril in combination with hydration and antibiotics, mitigated acute and delayed radiation injuries in the lungs and kidneys in rats (Fish et al., 2016). In a cell culture experiment, lisinopril suppressed the increase of Ang II in a medium of serum-starved endothelial cells (Wu et al., 2015). In terms of organs other than the lungs, it has been shown that lisinopril has anti-inflammatory effects on Freund’s adjuvant-induced arthritis in rats (Arab et al., 2022), and antioxidative as well as antifibrotic effects on cardiomyocytes (Scisciola et al., 2022).

In our study, as expected, lisinopril dramatically decreased Ang II and Ang 1-5 plasma levels. Total protein content and concentrations of inflammatory markers in BALF as well as the histological score for lung injury were decreased by lisinopril treatment, which indicated that lisinopril was able to attenuate VILI, while also causing hypotension.

The primary adverse effects of ACE inhibitors include hyperkalemia, dry cough, angioedema, dizziness, and renal insufficiency (Reid et al., 1993; Bezalel et al., 2015). Furthermore, the hypotensive effect of captopril impedes its clinical applicability for treatment or prevention of VILI (Jiang et al., 2007). This is especially of concern in critically ill patients requiring mechanical ventilation who are at particular risk for VILI. So far there is little published data on the way aminopeptidase inhibitors modify the RAS and whether they have any hemodynamic effects (Ahmad and Ward, 1990). Our results showed that the combination of lisinopril with ALT-00 at 10 and 100 μg/kg/min led to higher Ang II plasma levels, as compared to lisinopril alone. Therefore, we speculate that improved stabilization of Ang II by inhibiting its degradation by aminopeptidases resulted in sufficient vasoconstriction to keep the MAP in the same range as in untreated HVT mice. Ang II acts as a vasoconstrictor through Ang II type 1 receptors while Ang 1-7 induces vasodilation through the Mas receptor (Forrester et al., 2018). In this model we could not discern the individual effects on the MAP of increased Ang II and Ang 1-7 levels induced by the combination of lisinopril and ALT-00. What we observed in this study were the net effects on the MAP of a certain angiotensin metabolite profile in each treatment group, and we cannot exclude influences from other vasoactive mediators, such as bradykinin, that were not measured. As Ang II levels in the lisinopril + ALT-00 (10 and 100 μg/kg/min) groups were not higher than in the sham group, there was still a downregulation of Ang II compared to the HVT group without treatment.

Interestingly, we found that at 10 μg/kg/min of ALT-00, PRA-S (Ang I+Ang II) was increased without an increase in PRA, while at 100 μg/kg/min, PRA-S was increased together with PRA. This may also have been the reason why the highest Ang I and Ang 1-7 levels were found at the highest dose of ALT-00. Of note, the Ang II levels observed at the highest dose of ALT-00 were not higher than in the sham group, so that an additional negative feedback from Ang II levels on renin production was likely not present in this group. Although these findings may suggest that ALT-00 increased PRA, it is important to note that these results were obtained in absence of specific inhibitors and thus provide the sum of angiotensin metabolites under equilibrium conditions. Therefore, we cannot rule out that continued metabolism by various peptidases may have contributed to these findings. ACE activity in plasma (Ang II/Ang I ratio, ACE-S) was downregulated by lisinopril and significant suppression of ACE-S < 1 was maintained in combination with ALT-00 at all three dose levels. However, as we did not measure ACE activity directly, other enzyme activities may also have contributed to changes in ACE-S. The alternative RAS activation marker ALT-S was increased by ALT-00 at a dose of 100 μg/kg/min, most likely because of improved stabilization of Ang 1-7.

This study had five major limitations. First, lisinopril was only applied at a single dose level according to our previous pilot research. Since the current study identified strong effects of lisinopril on ACE-S and Ang II levels, we speculate that a lower dose of lisinopril in combination with ALT-00 may have been sufficient for beneficial effects on VILI, which in turn would have required a lower dose of ALT-00 to stabilize Ang II. Second, ALT-00 was not applied alone, which may be informative to elucidate its effects on hemodynamics and the angiotensin metabolite profile, but was not expected to show benefits in VILI. Third, we did not investigate the effects of lisinopril ± ALT-00 on tissue levels of angiotensin metabolites nor ACE and ACE2 protein expression or plasma levels. Fourth, we did not investigate the effects on bradykinin, which could also contribute to the observed blood pressure-normalizing effects, since ACE and aminopeptidases are involved in the degradation and generation of bradykinin respectively (Borghi et al., 2023). Fifth, major differences exist in basic angiotensin metabolite levels between mice and humans.

## 5 Conclusion

In summary, the combination of lisinopril with ALT-00 at dose levels of 10 and 100 μg/kg/min not only activated the anti-inflammatory alternative RAS pathway, but also prevented hypotension induced by lisinopril by a partial upregulation of Ang II plasma levels, and reduced the severity of VILI. As ALT-00 at a dose of 100 μg/kg/min showed unwanted side effects (increased PRA and no significant beneficial effects on inflammation markers), we conclude that the optimal ALT-00 dose for combination with lisinopril would be 10 μg/kg/min in order to counteract VILI without additional hypotension.

## Data Availability

The raw data supporting the conclusion of this article will be made available by the authors, without undue reservation.
