# Supplementary material for: Combined angiotensin-converting enzyme and aminopeptidase inhibition for treatment of experimental ventilator-induced lung injury in mice
Source: Front Physiol. 2023 Mar 30;14:1109452. doi: 10.3389/fphys.2023.1109452 (PMC10097933; doi:10.3389/fphys.2023.1109452)
Supplement: Supplementary file 1 [file Image2.pdf]

Supplemental Figure 2

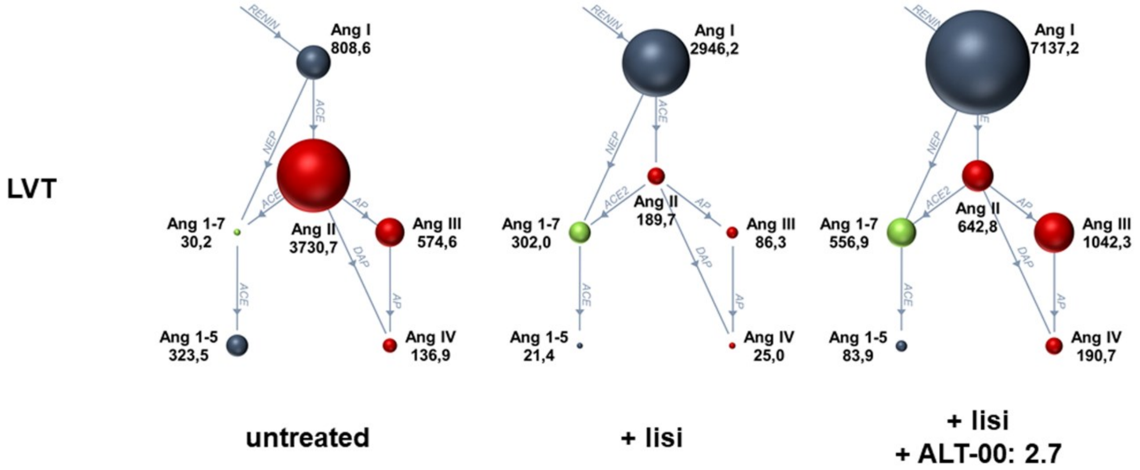

Suppl. Figure 2. Renin-angiotensin system Fingerprints from equilibrium analysis of plasma samples of each experimental group. The median plasma concentrations of angiotensin metabolites in pmol/L in the RAS Fingerprints correspond to the sphere sizes, n = 7/group. Ang: Angiotensin, LVT: low tidal volume, lisi: lisinopril, dose level of ALT-00 in µg/kg/min.
