# Supplementary material for: Combined angiotensin-converting enzyme and aminopeptidase inhibition for treatment of experimental ventilator-induced lung injury in mice
Source: Front Physiol. 2023 Mar 30;14:1109452. doi: 10.3389/fphys.2023.1109452 (PMC10097933; doi:10.3389/fphys.2023.1109452)
Supplement: Supplementary file 2 [file Image1.PDF]

Supplemental Figure 1

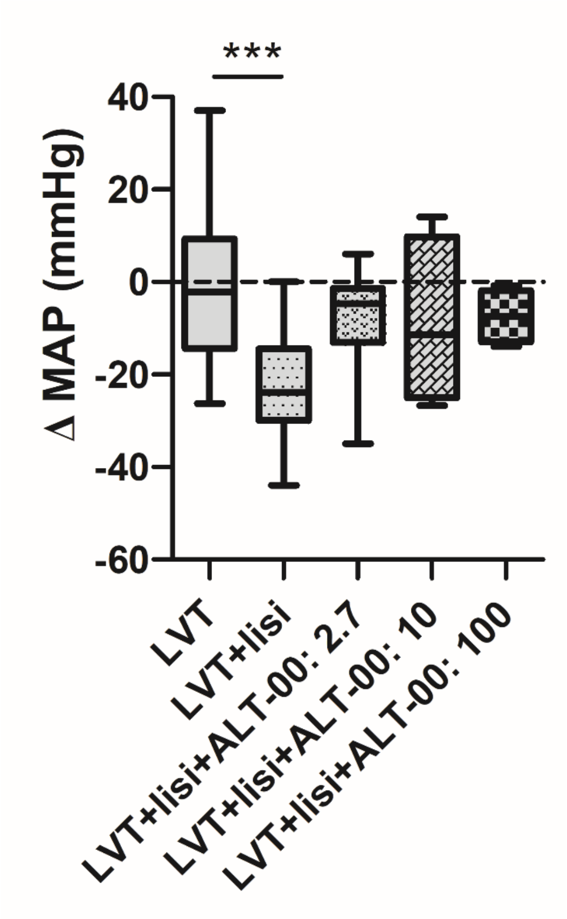

Suppl. Figure 1. Hemodynamic parameters after 4 hours of ventilation. (A) Mean arterial pressure (MAP): Low tidal volume (LVT) ventilation + lisinopril (lisi) infusion substantially decreased the MAP shown here as a negative ΔMAP (= MAP at 4 hours – MAP at baseline), which was prevented by additional treatment with ALT-00 at 2.7, 10 and 100 µg/kg/min. n = 14/14/13/4/4/group respectively, ***p < 0.001 compared by one-way analysis of variance with Tukey posttest.
